# TREM2: A Potential Target for the Targeted Therapy of Metabolic Diseases

**DOI:** 10.1155/mi/6210178

**Published:** 2026-09-09

**Authors:** Yaling Li, Sheng Quan Liu, Chun Chu, Jun Yang

**Affiliations:** ^1^ Department of Cardiology, The First Affiliated Hospital, Hengyang Medical School, University of South China, Hengyang, Hunan, China, usc.edu.cn; ^2^ Department of Pharmacy, The Second Affiliated Hospital, Hengyang Medical School, University of South China, Hengyang, Hunan, China, usc.edu.cn

**Keywords:** immunometabolism, macrophages, metabolic diseases, targeted intervention, TREM2

## Abstract

Metabolic diseases are characterized by profound dysregulation of the immunometabolic network, in which the interplay between chronic inflammation and metabolic disturbances form a self‐amplifying vicious cycle that drives disease progression. disease progression. Triggering receptor expressed on myeloid cells 2 (TREM2), a central regulator of immunometabolism, interacts with the adaptor protein DAP12 to orchestrate key macrophage functions including polarization, phagocytic clearance, inflammation resolution, and metabolic reprogramming. Through these processes, TREM2 plays a critical role in sensing metabolic stress signals and maintaining immunometabolic homeostasis. In this review, we systematically summarize recent advances in understanding the roles of TREM2 in major metabolic diseases, including atherosclerosis (AS), diabetes mellitus and its complications, obesity, and non‐alcoholic steatohepatitis (NASH), while highlighting its functional heterogeneity across different diseases contexts. We further dissect the regulatory mechanisms governing TREM2 activity, with particular emphasis on cell subset‐specific expression patterns, microenvironmental signal interactions, and post‐translational modifications (PTMs). Moreover, we summarize emerging TREM2‐targeted therapeutic strategies, including soluble TREM2 (sTREM2), agonistic antibodies, and traditional Chinese medicine (TCM)‐based approaches, and evaluate the translational potential of sTREM2 as a noninvasive biomarker. Emerging research frontiers involving TREM2, such as the gut microbiota–metabolism axis and interorgan communication, are also discussed. Finally, we identify key unresolved questions and future research priorities, providing a theoretical framework and potential strategies for precision diagnosis and targeted intervention in metabolic diseases.

## 1. Introduction

Metabolic diseases refer to a class of chronic disorders resulting from dysregulated metabolism of essential biomolecules, including carbohydrates, lipids, and purines [1]. Studies published in *Cell Metabolism* have clearly demonstrated that the incidence of metabolic diseases is increasing annually, with their mortality burden exceeding that of cancer and representing the second leading cause of death worldwide, following only cardiovascular diseases [2]. Currently recognized risk factors include genetic predisposition, an unhealthy diet, physical inactivity, chronic sleep deprivation, smoking, and alcohol consumption [3]. The pathogenesis of metabolic diseases is highly complex, and a well‐established hallmark of their development is the imbalance in immunometabolic networks, characterized by the intricate interplay between chronic low‐grade inflammation and metabolic dysregulation, which establishes a self‐perpetuating vicious cycle. Among these mechanisms, myeloid cell‐driven immune responses, particularly macrophage‐mediated processes, are increasingly recognized as critical regulators of metabolic homeostasis [4].

Among these, triggering receptor expressed on myeloid cells 2 (TREM2), a pivotal immunoregulatory molecule, has attracted increasing attention in recent years. TREM2 is predominantly expressed on the surface of myeloid lineage cells and participates in the pathogenesis of various diseases by regulating functions such as inflammation, phagocytosis, metabolism, and cell survival [5, 6]. Although TREM2 was initially primarily studied for its role in neurodegenerative diseases, accumulating evidence has revealed its essential roles in the initiation and progression of metabolic disorders. Increasing evidence indicates that TREM2 functions as a critical sensor of metabolic stress signals, including lipids and apoptotic debris, thereby orchestrating macrophage polarization, lipid metabolism, and inflammatory resolution and playing a pivotal role in maintaining immune–metabolic homeostasis [7]. However, emerging studies have also revealed context‐dependent effects of TREM2. For example, TREM2 has been reported to exacerbate sepsis by suppressing fatty acid oxidation in macrophages [8], highlighting that the net biological outcomes of TREM2 activation are highly dependent on the specific cellular and pathological microenvironments. For instance, in the context of obesity, sustained metabolic stress induces the cleavage and functional inactivation of the TREM2 extracellular domain (ECD), thereby impairing the ability of macrophages to eliminate lipid‐damaged hepatocytes and consequently exacerbating chronic hepatic inflammation and the progression of non‐alcoholic steatohepatitis (NASH) progression [9]. In diabetes‐induced kidney injury, enhancing TREM2 expression in renal macrophages ameliorates diabetes‐related renal damage [10]. Therefore, a comprehensive understanding of the molecular mechanisms underlying TREM2‐mediated regulation in metabolic diseases is of critical importance for elucidating disease initiation and progression, identifying novel therapeutic targets, and developing personalized treatment strategies. This review will systematically summarize the latest research progress on TREM2 in metabolism‐related diseases, explore its potential role in disease pathogenesis, and provide a theoretical and practical basis for future research and clinical applications.

## 2. Molecular Structure and Functions of TREM2 (Figure 1)

### 2.1. Expression Pattern, Genetic Characteristics, and Molecular Structure of TREM2

TREM2 is an extracellular innate immune receptor predominantly expressed on myeloid lineage cells, including dendritic cells (DCs) and tissue‐resident macrophages (including osteoclasts and microglia) [5]. As a member of the TREM family of transmembrane glycoproteins, TREM2 belongs to the immunoglobulin variable (IgV) domain receptor family. Human TREM2 gene is located on chromosome 6p21.1, whereas the murine ortholog is located on chromosome 17C3, this genomic region also contains genes encoding other members of the TREM family, including TREM‐1, TREM‐3 (in mice), and “TREM‐like” genes [11]. TREM2 is a glycoprotein with an approximate molecular weight of 40 kDa, which is reduced to approximately 26 kDa following N‐deglycosylation. Structurally, TREM2 consists of an ECD, a transmembrane region, and a short cytoplasmic tail. The ECD, encoded by exon 2, contains a single V‐type immunoglobulin superfamily (Ig‐SF) domain with three potential N‐glycosylation sites. The transmembrane region contains a conserved charged lysine residue, whereas the cytoplasmic tail lacks signaling motifs. Therefore, TREM2 requires association with an auxiliary signaling adaptor protein to initiate downstream signal transduction upon receptor activation [12–14].

**Figure 1:**
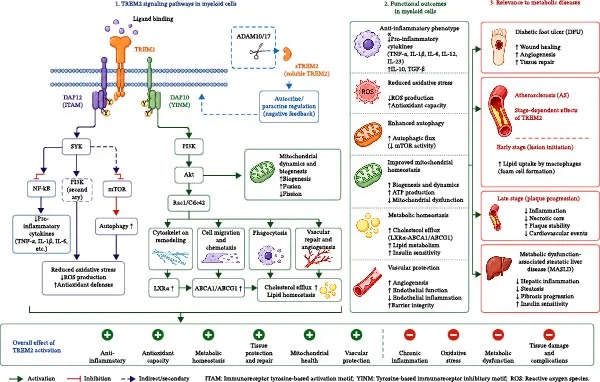
TREM2 signaling in myeloid cells and its roles in metabolic diseases. Left panel: TREM2 signals through DAP12/DAP10, activating SYK and PI3K/Akt pathways to regulate inflammation, phagocytosis, migration, cholesterol efflux, and autophagy. ADAM10/17‐mediated shedding releases soluble TREM2 (sTREM2). Middle panel: TREM2 activation promotes anti‐inflammatory responses, antioxidant defense, autophagy, mitochondrial function, lipid metabolism, and insulin sensitivity. Right panel: TREM2 exhibits stage‐ and context‐dependent effects in metabolic diseases: promotes repair in DFU, regulates plaque progression in AS (early foam cell formation and late plaque stabilization), and alleviates inflammation in MASLD. Bottom panel: TREM2 regulates inflammation, oxidative stress, metabolic homeostasis, and tissue injury. Green arrows indicate activation; red blunt lines indicate inhibition; and dashed lines indicate indirect effects. ROS, reactive oxygen species.

### 2.2. Ligands and Signaling Pathways of TREM2

TREM2 is a receptor that interacts with a diverse array of ligands, many of which serve as indicators of tissue damage and cellular stress [6]. Its ligands include a broad range of anionic molecules, either in soluble or membrane‐associated forms, such as bacterial products, DNA, lipoproteins, and phospholipids [5]. Although the cytoplasmic tail of TREM2 lacks intrinsic signaling motifs, oppositely charged residues in its C‐terminal transmembrane region can interact with the 12 kDa adaptor protein TYROBP (also known as the DNAX‐activating protein of 12 kDa, DAP12) or DAP10 [13, 14]. DAP12 is a type I transmembrane adaptor protein containing an immunoreceptor tyrosine‐based activation motif (ITAM) within its cytoplasmic domain. In humans, the DAP12 gene is located on chromosome 19q13.1 and facilitates downstream activation of spleen tyrosine kinase (Syk). In contrast, DAP10 propagates signals by recruiting phosphatidylinositol 3‐kinase (PI3K) [15]. Following TREM2‐ligand interaction, these coreceptors are phosphorylated and recruit intracellular signaling machineries, which further regulate signaling pathways such as NF‐κB, PLCγ2, and Akt signaling cascades. Through these mechanisms, TREM2 participates in multiple cellular functions, including cell differentiation, survival, phagocytosis, inflammatory responses, and metabolic reprogramming.

### 2.3. Functions of TREM2

#### 2.3.1. Promotion of Cell Differentiation and Survival

Microglia play critical roles in the response to neural injury and the pathogenesis of neurodegenerative diseases. TREM2 is a key regulator of microglial proliferation and survival, potentially through the activation of the Wnt/β‐catenin signaling pathway. β‐Catenin, a core component of the canonical Wnt pathway, is essential for maintaining diverse biological processes, including cellular survival and homeostasis [6, 16]. TREM2 activation enhances anti‐apoptotic signaling to prevent premature death of immune cells; it may also protect neurons from apoptosis through activation of the PI3K/Akt pathway [17]. In the presence of TGF‐β1, TREM2 deficiency has been reported to promote both M1 and M2 macrophage polarization through the JAK–STAT pathway, consequently impairing macrophage survival and facilitating apoptosis via downregulation of Akt/mTOR signaling [18]. Additionally, deficiency of the DAP12/TREM2 signaling axis has been shown to impair osteoclast differentiation and functional activity [19, 20].

#### 2.3.2. Regulation of Phagocytosis

TREM2 plays a pivotal role in regulating phagocytic activity. Impaired or complete knockout of TREM2 significantly reduces the phagocytic uptake and clearance of various substrates, including β‐amyloid (Aβ), apoptotic cells, bacteria, lipoproteins, and cellular debris, whereas TREM2 activation or overexpression enhances the phagocytic capacity. This function is particularly critical in neurodegenerative diseases, such as Alzheimer’s disease (AD), where the accumulation of pathological Aβ plaques induces neuroinflammation and neuronal damage [6, 21–23]. The mechanisms underlying TREM2‐dependent phagocytosis are complex and context‐dependent and remain incompletely characterized. Current evidence suggests that these mechanisms may involve cytoskeletal remodeling and kinase activation in specific cellular contexts, while displaying considerable variation among different cell types and experimental conditions [24].

#### 2.3.3. Inhibition of Inflammation

In innate immune responses, TREM2 is regarded as an important negative regulator of inflammatory activation. TREM2 activation has been implicated in the regulation of inflammatory responses through signaling pathways including PI3K/Akt and nuclear factor kappa B (NF‐κB). The PI3K/Akt pathway plays essential roles in regulating cell survival, proliferation, and inflammatory responses, while the NF‐κB pathway serves as a central mediator in numerous immune processes, whereas the NF‐κB pathway serves as a central mediator of diverse immune processes, particularly inflammation. Through modulation of these signaling cascades, TREM2 activation may indirectly promote the production of anti‐inflammatory cytokines or suppress the release of pro‐inflammatory cytokines [17, 25, 26]. Furthermore, TREM2 activation can attenuate inflammatory responses by reducing neutrophil infiltration, downregulating the expression of pro‐inflammatory mediators (e.g., tumor necrosis factor‐α [TNF‐α] and interleukin‐1β [IL‐1β]), modulating immune cell morphology and phenotypic states, and suppressing complement component 1q (C1q) production in alveolar macrophages (AMs) [25, 27].

#### 2.3.4. Regulation of Metabolism

Accumulating evidence indicates that TREM2 serves as an important regulator of lipid metabolism. In the central nervous system (CNS), TREM2 acts as a lipid receptor to regulate the metabolism of cholesterol, phospholipids, and myelin components, facilitate the transition of microglia toward a disease‐associated phenotype, and interact with apolipoprotein E (ApoE). Peripherally, TREM2 influences lipid metabolism by regulating the development and progression of obesity and its associated complications and may link central and peripheral lipid metabolism by affecting blood–brain barrier (BBB) integrity [28, 29]. TREM2 deficiency disrupts glycolytic activity and oxidative metabolism in Schwann cells (SCs); the resulting energy imbalance may induce mitochondrial dysfunction and autophagy through the activation of AMPK signaling and inhibition of the PI3K/Akt/mTOR pathway [30].

## 3. TREM2 and Metabolism Disease (Table 1)

### 3.1. TREM2 and Atherosclerosis (AS)

AS is an arterial wall disease caused by hyperlipidemia, vascular inflammation, and immune dysregulation [31]. Its key features include chronic inflammation, abnormal immune responses, and disturbances in key lipid metabolic processes [32]. Foam cells play a critical role in the pathogenesis of AS: following the deposition of oxidized low‐density lipoprotein cholesterol (ox‐LDL‐C) in the intima of blood vessels, monocyte‐derived macrophages or smooth muscle cells migrate to the arterial intima, phagocytose ox‐LDL‐C, and transform into foam cells. These foam cells undergo programmed cell death through multiple pathways, leading to the formation of a necrotic core in atherosclerotic plaques. Subsequent development of lipid streaks and fibrous plaques ultimately results in vascular lumen stenosis and rupture [33, 34]. In recent years, single‐cell RNA sequencing and other high‐throughput approaches have revealed that TREM2 is highly expressed in foam macrophage populations within atherosclerotic plaques [35, 36]. Notably, TREM2 appears to exert context‐dependent effects in AS, exhibiting both protective functions and potential pro‐atherogenic activities.

**Table 1:** Evidence for an important role of the TREM2 in metabolic disease.

Disease	Cell types	TREM2 expression characteristics	Downstream signaling pathways	Disease stage specificity	TREM2 mechanism of action	Therapeutic implications	References
AS	Foam macrophages (monocyte‐derived); vascular smooth muscle cells	High expression in foam macrophage clusters within atherosclerotic plaques	DAP12/Syk/PI3K/Akt/mTOR; CD36; LXR‐mediated cholesterol efflux; OXPHOS reprogramming	Early lesions: pro‐atherogenic (promotes lipid uptake and plaque expansion); Advanced lesions: plaque‐stabilizing (enhances cholesterol efflux, collagen deposition, reduces necrotic core)	Stage‐specific dual function: pro‐atherogenic in early phases via promoting foam macrophage survival and lipid uptake; plaque‐stabilizing in advanced stages via enhancing efferocytosis and reducing inflammation	TREM2 agonist antibodies (4D9, AL002a) for advanced plaques to enhance stability; Geniposide inhibits TREM2/mTOR axis for early lesions; Timing of intervention is critical; Circulating sTREM2 as biomarker for plaque progression	[31–40]
T1D	Islet‐infiltrating macrophages	Expression in islet‐infiltrating macrophages	NF‐κB (inhibition); PI3K/Akt	Early autoimmune insulitis phase: protective (clearing apoptotic β‐cell debris to suppress autoimmune amplification)	Protective roles: Phagocytosing apoptotic cell debris to suppress autoimmune amplification; exerting anti‐inflammatory effects via inhibiting TNF‐α and IL‐1β production	TREM2 activation to maintain immune tolerance and delay autoimmune progression; potential for preventing β‐cell destruction	[41–46]
T2D	Adipose tissue macrophages (F4/80^+^CD11c^+^ pro‐inflammatory subset); pancreatic islet macrophages	Expression in both adipose tissue and pancreatic islet macrophages	NF‐κB/CXCL3 axis; PI3K/Akt; TLR4 (antagonistic interaction)	Early: modulating adipose tissue remodeling and suppressing local inflammation; progressive: promoting M1‐to‐M2 polarization to protect β‐cells	Protective roles: modulating adipose tissue remodeling, suppressing local inflammation, and improving insulin sensitivity; promoting macrophage M1‐to‐M2 polarization, thereby protecting β‐cells	Targeting TREM2 to improve insulin sensitivity; sTREM2 as potential therapeutic agent for inflammation resolution	[25, 47]
DN	Macrophages in glomerular mesangial region and renal tubulointerstitium; TREM2^+^ macrophages; renal tubular epithelial cells	Highly expressed on macrophages in glomerular mesangial region and renal tubulointerstitium; Urinary TREM2^+^ macrophage levels negatively correlate with eGFR	DAP12/Syk; NF‐κB; Smad3 (phosphorylation inhibition); CD36; IL‐1β‐mediated pathway	Progressive: expression correlates with renal inflammation and fibrosis degree; TREM2^+^ macrophages expand with disease progression	Protective roles: Inhibiting macrophage M1 polarization and attenuating renal inflammation; delaying renal interstitial fibrosis; reducing lipid accumulation and ferroptosis in renal tubular epithelial cells	Targeted TREM2 activation as novel therapeutic basis for DN; Urinary TREM2^+^ macrophages as biomarker for DN progression and severity assessment	[10, 48]
DR	Retinal Müller glial cells; M2 macrophages (migrasome‐producing)	Expression elevated in retinas of diabetic mice; enhanced ribosome binding of TREM2 and DAP12, CSF1R mRNAs in retinal Müller glial cells	DAP12; CSF1R; TREM2/DAP12 pathway; IL‐12‐mediated activation	Early: protective (alleviates inflammatory injury, promotes microglial phagocytosis); advanced: pathogenic (mediates pathological neovascularization via M2 macrophage‐derived migrasomes)	Dual roles: pathogenic role—mediating pathological neovascularization through M2 macrophage‐derived migrasomes; protective role—alleviating early retinal inflammatory injury via TREM2/DAP12 pathway	Stage‐specific intervention strategy required; IL‐12 activation of TREM2/DAP12 pathway for early neuroprotection; TREM2 inhibition may be needed for advanced neovascularization	[49–51]
DFU	Wound‐associated macrophages; dermal fibroblasts; mesenchymal stem cells	Expression in wound‐associated macrophages; upregulated following wound induction in diabetic model	IL‐4/TREM2 (nonclassical receptor); MAPK/AP‐1 (inactivation); DAP12	Healing phase: protective (promotes M2 polarization, angiogenesis, collagen deposition, and tissue repair)	Protective roles: modulating macrophage M2 polarization to attenuate inflammation; promoting angiogenesis and collagen deposition; enhancing tissue repair capacity; mediating macrophage‐fibroblast crosstalk via IL‐4/TREM2 axis	sTREM2 as endogenous TREM2 agonist for wound healing promotion; IL‐4/TREM2 axis as viable therapeutic target; macrophage‐specific TREM2 targeting for DFU treatment	[52–54]
Obesity	Lipid‐associated macrophages (LAMs); visceral adipose tissue macrophages; F4/80^+^CD11c^+^ inflammatory macrophages	Highly expressed in visceral adipose tissue LAMs; sex‐specific associations with BMI (males: expression‐BMI correlation; females: rs2234256 SNP effect)	Wnt10 b/β‐catenin (inhibition); DAP12; adipogenesis‐related genes	Early/physiological: maintains adipose tissue metabolic homeostasis; overexpression/late: promotes obesity and insulin resistance	Dual roles: physiological role—maintaining adipose tissue metabolic homeostasis via functional LAMs; pathological role—promoting obesity and insulin resistance when overexpressed via inhibiting Wnt10 b/β‐catenin pathway	Gender‐specific targeting strategies; tissue TREM2 expression as stratification biomarker for obesity‐related metabolic disorders; modulating LAM function for metabolic homeostasis	[55–58]
NAFLD/NASH	Kupffer cells (resident); monocyte‐derived macrophages (Mo‐Mφ); TREM2^+^ macrophage subset	TREM2^+^ macrophage subset in liver; mRNA upregulation with protein depletion phenomenon in NASH progression	DAP12; MS4A7; NLRP3 inflammasome (suppression); S1P/S1PR1 (transcriptional upregulation); ADAM17 (protein cleavage)	Early NASH: protective (efferocytosis of apoptotic hepatocytes, anti‐inflammatory via IL‐10 release); Late NASH: TREM2 protein depletion by ADAM17‐mediated cleavage, loss of protective function, disease exacerbation	Bifunctional regulation: early phase—phagocytosing apoptotic hepatocytes and exerting anti‐inflammatory effects; late phase—TREM2 protein depletion impairs efferocytosis, forming vicious cycle of inflammatory amplification driving fibrosis	S1PR1 agonist (FTY720) to upregulate TREM2 transcription; ADAM17 inhibition to prevent TREM2 cleavage; sTREM2 as diagnostic biomarker (exclusion threshold 38 ng/mL, inclusion threshold 65 ng/mL for NASH); reducing liver biopsy need	[9, 59]

On one hand, in early lesions, TREM2 promotes lipid uptake and contributes to the enlargement of atherosclerotic plaques. The underlying mechanisms may involve TREM2‐mediated activation of Syk, regulation of scavenger receptor CD36, modulation of other lipid‐handling signaling molecules, or activation of liver X receptors (LXRs) to enhance cholesterol efflux, thereby supporting the proliferation and survival of foam macrophages. This sustained macrophage lipid uptake, in the absence of effective cell death‐mediated clearance, contributes to lesion expansion, and TREM2 inhibition has been shown to slow AS progression [36–38]. On the other hand, despite increasing plaque size, TREM2 may promote plaque stability during the advanced stages of AS through multiple mechanisms. These include restoring foam macrophage functions to facilitate cholesterol efflux and collagen deposition, limiting necrotic core formation, and reprogramming macrophage metabolism toward oxidative phosphorylation (OXPHOS). Additionally, TREM2 can enhance macrophage activity to clear accumulated necrotic debris within plaques and reduce pro‐inflammatory factors, thereby attenuating inflammation [30, 36, 39]. Notably, as summarized in a recent review by Zhu et al. [39], Piollet et al. [30] demonstrated the stage‐dependent effects of TREM2 in AS using two complementary experimental approaches: hematopoietic TREM2 deficiency increased necrotic core formation during early lesion development, while TREM2 agonistic antibody 4D9 showed limited efficacy in reducing necrotic core size in advanced lesions. Xu et al. [40] reported that TREM2 overexpression in RAW264.7 macrophages suppresses autophagy through activation of mTOR signaling, whereas geniposide inhibits the TREM2/mTOR signaling axis and enhances autophagic activity in plaque macrophages, potentially attenuating atherosclerotic progression. The dual role of TREM2 in AS is likely associated with its functional heterogeneity among distinct immune cell subsets, dynamic alterations in the local microenvironment, and complex interactions with other cytokines and signaling factors.

### 3.2. TREM2 and Diabetes Mellitus

Diabetes mellitus is a complex metabolic disease characterized by chronic hyperglycemia, primarily classified into type 1 diabetes (T1D) and type 2 diabetes (T2D). T1D results from the destruction of pancreatic β‐cells by autoimmune responses, leading to absolute insulin deficiency [60]. In contrast, T2D is closely associated with insulin resistance and progressive pancreatic β‐cell dysfunction [61]. Chronic low‐grade inflammation serves as a core pathological link connecting obesity, insulin resistance, and T2D, wherein the infiltration and activation of immune cells such as macrophages in metabolic organs (e.g., pancreas, adipose tissue, and liver) play pivotal roles [62]. In recent years, TREM2, as a key regulator of macrophage function, has exhibited distinct yet critical roles in both types of diabetes and their complications.

In T1D, autoimmune insulitis is a hallmark feature, targeting insulin‐secreting cells. We note at the outset that direct experimental evidence linking TREM2 to autoimmune diabetes is still lacking; the protective role proposed below is inferred largely from studies in other diseases and should therefore be regarded as a working hypothesis. Autoimmune β‐cell destruction generates extensive apoptotic debris within the islets. Failure to promptly clear this debris leads to secondary necrosis, releasing intracellular antigens that exacerbate local inflammation and activate additional autoimmune responses [41–43]. Notably, in neurological and liver diseases, TREM2‐expressing macrophages have been shown to efficiently phagocytose apoptotic cells and debris, promote inflammation resolution, and maintain immune tolerance—thus potentially delaying or inhibiting the progression of autoimmune responses [44, 45]. Additionally, studies indicate that TREM2 activation can reduce the production of pro‐inflammatory factors, such as TNF‐α and IL‐1β, by inhibiting pro‐inflammatory signaling pathways including NF‐κB [46]. By analogy, one might expect TREM2 to limit the magnitude of autoimmune destruction in the pancreas through the same “clearance” and “anti‐inflammatory” mechanisms. However, whether this expectation holds in the diabetic islet microenvironment, where cytokine stress and glucotoxicity modify macrophage behavior remains to be tested directly in T1D models. In T2D, TREM2 primarily exerts its effects by regulating adipose tissue inflammation, improving insulin resistance, and preserving pancreatic β‐cell function. Liu et al. [47] established wild‐type and TREM2‐deficient mouse models subjected to a high‐fat diet (HFD) and demonstrated that TREM2 deficiency resulted in excessive infiltration of F4/80^+^CD11c^+^ pro‐inflammatory macrophages into adipose tissue, accompanied by adipocyte hypertrophy, increased apoptosis, and significantly elevated levels of adipose tissue inflammatory factors (e.g., TNF‐α and IL‐1β). These inflammatory factors directly interfere with the insulin signaling pathway, leading to more severe insulin resistance in mice. In contrast, maintained TREM2 expression preserves insulin sensitivity by promoting adipose tissue remodeling and suppressing local inflammatory responses, providing critical evidence that TREM2 alleviates adipose tissue inflammation to improve insulin resistance [47]. In T2D pancreatic tissue, macrophage infiltration‐induced inflammation promotes β‐cell apoptosis and impairs insulin secretion. Notably, studies have reported that TREM2 regulates the NF‐κB/CXCL3 signaling axis to facilitate macrophage polarization from the pro‐inflammatory M1 phenotype toward the anti‐inflammatory M2 phenotype, thereby ameliorating the pathological state of inflammatory infiltration [25]. We, therefore, hypothesize that TREM2 may alleviate pancreatic β‐cell apoptosis by promoting macrophage M1‐to‐M2 polarization; however, this possibility has not yet been directly examined in the diabetic pancreas and requires a dedicated investigation.

### 3.3. TREM2 and Diabetic Complications

Recent studies have demonstrated that TREM2 also plays important regulatory roles in diabetic microvascular complications (e.g., diabetic nephropathy [DN] and diabetic retinopathy [DR]) and macrovascular complications (e.g., diabetic cardiomyopathy [DCM] and diabetic foot disease). In DN, Subramanian et al. [10] analyzed clinical data from DN patients and found that the number of TREM2‐positive macrophages in the kidney tissue was negatively correlated with estimated glomerular filtration rate (eGFR) and urinary albumin‐to‐creatinine ratio (UACR), suggesting TREM2 as a potential biomarker for DN progression. In renal tissues from DN patients and db/db diabetic mice, TREM2 is primarily expressed on the surface of macrophages in the glomerular mesangial area and the renal tubulointerstitium, with its expression level positively correlated with the degree of renal inflammation and fibrosis [10]. Furthermore, this study confirmed that TREM2 activation inhibits the polarization of renal macrophages toward the pro‐inflammatory M1 phenotype via the DAP12/Syk pathway, reducing the release of TNF‐α, IL‐1β, and chemokines, alleviating local inflammatory infiltration in renal tissues, and mitigating the toxic damage of inflammatory factors to glomerular podocytes and renal tubular epithelial cells. TREM2 activation also attenuates the progression of renal interstitial fibrosis by inhibiting the NF‐κB pathway and Smad3 phosphorylation to reduce the release of profibrotic factors, whereas TREM2 deficiency leads to excessive secretion of profibrotic factors by renal macrophages, promoting fibroblast activation and collagen deposition [10]. Additionally, Wang et al. [48] showed that TREM2^+^ macrophages exert a protective effect against DN by inhibiting IL‐1β‐mediated CD36 expression in renal tubular epithelial cells, thereby reducing lipid accumulation and ferroptosis. Collectively, targeted activation of TREM2 may provide a novel theoretical basis for the treatment of DN.

DR is a leading cause of blindness among adults, with its pathogenesis primarily involving retinal vascular endothelial dysfunction, inflammatory infiltration, and abnormal angiogenesis [49]. Studies using STZ‐induced diabetic mouse models enhanced the ribosome association of TREM2 and its related signaling pathway transcripts, including DAP12 and CSF1R, in retinal Müller glial cells. Quantitative PCR further verified a significant increase in TREM2 mRNA abundance in retinal immunoprecipitation samples, providing direct evidence for TREM2 expression in the retinas of STZ‐induced diabetic mice. Li et al. [50] reported that M2 macrophage‐derived migrasomes promote ischemia‐induced retinal angiogenesis through TREM2 targeting. During DR progression, retinal ischemia and hypoxia occur due to vascular damage, which may increase the production of migrasomes by M2 macrophages, promoting pathological angiogenesis that further disrupts the retinal structure and exacerbates DR.

In contrast, a recent study demonstrated that IL‐12 activates microglial phagocytosis via the TREM2/DAP12 pathway, thereby ameliorating diabetic retinal neurodegeneration [51]. These opposing findings may be attributed to differences in DR stage, TREM2 activation intensity, and target cell types, warranting further stratified investigations. Currently, the role of TREM2 in DR remains incompletely understood and controversial. Some studies suggest that TREM2 deficiency attenuates retinal angiogenesis, whereas others confirm that TREM2 activation alleviates early‐stage retinal inflammatory injury. These seemingly discrepancy may be attributed to differences in disease stage, the extent of TREM2 activation, and the specific target cell populations involved. Therefore, further stratified investigations are required to elucidate the context‐dependent functions of TREM2 across different phases of DR progression.

Diabetic foot ulcer (DFU) is one of the most common complications in diabetic mellitus, characterized by chronic and nonhealing ulcers of the lower extremities. Diabetic peripheral neuropathy, peripheral vascular disease, trauma, and excessive plantar pressure are the main causes of DFU [52, 53]. Skin wound healing is a complex physiological process involving the interactions among multiple tissue‐resident cells, immune cells, and regulatory factors. TREM2‐mediated macrophage signaling pathways not only regulate myeloid cell functions but may also influence the activity of nonmyeloid cells, including fibroblasts. For example, activating TREM2‐mediated signaling promotes mesenchymal stem cell differentiation and enhances tissue repair capacity; regulating lung fibroblast function accelerates post‐injury lung repair [63, 64]. Additionally, studies have indicated that TREM2 gene mutations impair macrophage phagocytosis of apoptotic cells, cellular debris, bacteria, and foreign factors, while TREM2 overexpression induces macrophage polarization toward the M2 phenotype, enhancing anti‐inflammatory and tissue repair effects [65, 66]. Zhu et al. [54] established a macrophage‐specific TREM2 knockout mouse model in diabetic wound tissue, which exhibited exacerbated inflammation, reduced angiogenesis, delayed wound healing, and decreased collagen deposition. Furthermore, in vivo administration of soluble TREM2 (sTREM2) effectively attenuated inflammatory responses and accelerated diabetic wound healing. In summary, TREM2 plays a critical protective role in DFU wound healing by regulating macrophage polarization, inflammation resolution, and nonmyeloid cell repair function, providing an important theoretical basis for the targeted treatment of DFU.

DCM is one of the most severe cardiovascular complications associated with T2DM. Its pathological progression is not merely mediated by direct hyperglycemia‐induced injury but rather involves a complex network of pathological processes, including chronic low‐grade inflammation, oxidative stress imbalance, myocardial remodeling (including fibrosis and hypertrophy), and metabolic reprogramming disorders [67]. Among these mechanisms, dysregulated immune activation, particularly macrophage‐mediated inflammatory responses, is considered a critical initiating event contributing to DCM development. Under diabetic conditions, infiltrating macrophages in the myocardial tissue polarize toward the pro‐inflammatory M1 phenotype, releasing large amounts of inflammatory factors, such as TNF‐α and IL‐6, which induce cardiomyocyte injury, endothelial dysfunction, and promote fibroblast proliferation, ultimately leading to impaired cardiac systolic/diastolic function [68]. We emphasize, however, that direct evidence linking TREM2 to DCM pathogenesis is currently lacking: no study to date has examined TREM2 expression or function specifically in diabetic myocardial tissue. The proposed link rests on two lines of indirect evidence. First, TREM2 is a well‐established regulator of macrophage polarization, inflammation resolution, lipid metabolism, and tissue repair [69], processes that overlap extensively with the pathological network of DCM. Second, in a myocardial infarction model, TREM2‐positive macrophages were shown to promote cardiac repair by reprogramming metabolism via SLC25A53 [70], demonstrating that TREM2 can influence cardiac tissue remodeling. Whether these mechanisms operate in the diabetic heart remains unknown. Several key questions therefore remain open: how does TREM2 expression change in the myocardial tissue under diabetic conditions? Does it affect DCM pathological progression by regulating specific signaling pathways? We regard TREM2 as a promising candidate target for DCM, but one whose feasibility must be validated directly in DCM animal models and clinical samples before therapeutic conclusions can be drawn.

### 3.4. TREM2 and Obesity

Obesity is a globally prevalent metabolic disorder characterized by excessive adipose tissue expansion and accumulation, which triggers chronic inflammation and metabolic abnormalities [55]. Recent studies have identified TREM2 as an important immunometabolic regulator involved in the initiation and progression of obesity. Clinical population studies have confirmed a close association between TREM2 expression and obesity‐related metabolic traits, with notable sex‐specific patterns. Analysis of GTEx database samples revealed a significant correlation between TREM2 expression in adipose tissue and BMI in males but not in females. In the UK Biobank cohort study, the rs2234256 genetic polymorphism of TREM2 exerted a significant effect on BMI in females, with both heterozygous and homozygous carriers showing significantly increased BMI [56]. These findings suggest a sex‐dependent role of TREM2 in the regulation of human obesity susceptibility and metabolic phenotypes.

In mouse obesity models, single‐cell sequencing has identified lipid‐associated macrophages (LAMs) with high TREM2 expression in the visceral adipose tissue, which are critical for maintaining adipose tissue metabolic homeostasis. TREM2 knockout prevents macrophages from forming functional LAMs, leading to adipocyte hypertrophy, body fat accumulation, glucose intolerance, and other obesity‐related metabolic disorders [57]. Furthermore, in an oleic acid‐induced high‐fat cellular model, activation of the TREM2 signaling pathway in macrophages markedly reduced lipid accumulation and triglyceride levels in cocultured hepatocytes, whereas blockade of TREM2 signaling abolished these regulatory effects [9]. Interestingly, Park et al. [58] conducted experiments using transgenic mice and found that TREM2 transgenic mice were more prone to obesity under HFD feeding, accompanied by adipocyte hypertrophy, insulin resistance, and hepatic steatosis. The underlying mechanism may involve TREM2 promoting obesity by regulating adipogenesis‐related genes and inhibiting the Wnt10 b/β‐catenin pathway, providing additional evidence for the involvement of TREM2 downstream signaling in obesity regulation [58]. Collectively, these findings highlight the dual and context‐dependent roles of TREM2 in obesity. While TREM2‐mediated LAM formation and lipid handling may exert protective effects by preserving metabolic homeostasis, excessive or dysregulated TREM2 activation may facilitate adipose expansion and metabolic deterioration under certain conditions. Therefore, the functional outcome of TREM2 signaling appears to depend on factors such as the cellular context, disease stage, metabolic environment, and the intensity of pathway activation.

### 3.5. TREM2 and NASH

NASH is a severe stage of non‐alcoholic fatty liver disease (NAFLD), characterized by the pathological triad of hepatic steatosis, chronic inflammation, and fibrosis. Abnormal activation of liver macrophages is a key initiating factor driving disease progression. Recent studies have confirmed that TREM2, as a core regulator of macrophage function, acts as a “bidirectional regulatory hub” in NASH pathogenesis by modulating efferocytosis, inflammation resolution, and metabolic reprogramming. The dysfunction of TREM2 is an important mechanism underlying NASH progression [71].

In early NASH, extracellular lipid droplets and apoptotic debris released by injured steatotic hepatocytes induce TREM2 upregulation in liver macrophages, forming a TREM2^+^ macrophage subset. By binding to the adaptor protein DAP12, these cells activate downstream signaling to (1) efficiently phagocytose apoptotic hepatocytes (efferocytosis), preventing secondary necrosis and antigen exposure caused by debris accumulation, thereby inhibiting the initiation of chronic inflammation at the source [9] and (2) synergize with MS4A7 to suppress NLRP3 inflammasome activation and the secretion of pro‐inflammatory factors, such as IL‐1β, while promoting the release of the anti‐inflammatory factor IL‐10, maintaining hepatic immune homeostasis [7].

Notably, NASH progression is characterized by a unique “TREM2 mRNA upregulation with protein depletion” phenomenon, which is a key mechanism underlying the loss of TREM2’s protective function and disease exacerbation. In liver tissues from human NASH patients and mouse models, Trem2 mRNA levels are persistently elevated, while TREM2 protein expression is drastically reduced, with almost no TREM2‐positive signals detected in core macrophage subsets such as Kupffer cells (KCs) [9]. This contradiction arises from the imbalance between “protective upregulation” and “inflammatory cleavage”: sphingosine‐1‐phosphate (S1P) released by apoptotic hepatocytes binds to macrophage S1PR1 receptors, initiating a compensatory mechanism of TREM2 transcriptional upregulation. However, persistently elevated pro‐inflammatory factors (e.g., TNF‐α and IL‐1β) in NASH activate the metalloproteinase ADAM17, which specifically cleaves TREM2 protein on the macrophage surface‐an effect not antagonized by S1P‐mediated upregulation [9]. TREM2 protein depletion directly impairs macrophage efferocytosis, leading to accumulation of apoptotic hepatocytes and further exacerbation of inflammation, forming a vicious cycle of inflammatory amplification that ultimately drives the progression of steatohepatitis to cirrhosis or even hepatocellular carcinoma. A recent study published in *Hepatology* analyzed a large number of liver biopsies and revealed that sTREM2 can serve as a biomarker for monitoring liver fibrosis progression in NAFLD. Compared with other NAFLD/NASH biomarkers, such as TIMP1, TIMP2, and MMP2, sTREM2 more accurately reflects NAFLD progression, suggesting its greater clinical application potential [59].

## 4. Core Driving Mechanisms of TREM2 Functional Heterogeneity

### 4.1. Cell Subset‐Specific Regulatory Mechanisms

Within the same tissue microenvironment, TREM2 expression and function exhibit substantial heterogeneity among distinct macrophage subsets. Differences in their cellular origins and molecular regulatory networks constitute a key basis for TREM2 functional differentiation in metabolic diseases. In liver tissue, KCs—as resident macrophages,undergo S1P‐induced transcriptional upregulation of TREM2 following exposure to apoptotic hepatocyte‐derived signals during the early stage of NASH. However, under chronic metabolic stress, increased KC apoptosis and impaired self‐renewal capacity result in rapid depletion of TREM2 protein through ADAM17‐mediated ectodomain shedding, leading to the near absence of detectable TREM2‐positive signals [9]. In contrast, monocyte‐derived macrophages (Mo‐Mφ), as recruited macrophage populations, continuously accumulate within the liver during NASH progression. These cells exhibit relatively enhanced TREM2 protein stability and activate efferocytosis‐related pathways through DAP12‐dependent signaling, thereby becoming the core force for hepatic inflammation resolution [9, 72]. In adipose tissue, LAMs, a specialized TREM2‐high macrophage population, maintain metabolic homeostasis by inhibiting the Wnt10 b/β‐catenin pathway, while inflammatory macrophages (e.g., F4/80^+^CD11c^+^ subsets) show significantly lower TREM2 expression and primarily exert pro‐inflammatory functions [57]. Collectively, this macrophage subset‐specific TREM2 expression pattern and functional differentiation specialization highlight the critical role of tissue microenvironments in shaping immune cell states, providing an important cellular and biological basis for deciphering TREM2’s heterogeneous roles in different metabolic diseases.

### 4.2. Microenvironmental Signal Crosstalk Mechanisms

Under different metabolic stress conditions, TREM2 forms complex cross‐regulatory networks with immune receptors and cytokines in the local microenvironment, determining the selective activation of its downstream signaling pathways. Ligand identity and availability constitute an additional layer of context‐dependence: TREM2 recognizes diverse anionic ligands, including lipoproteins, phospholipids, and apoptotic cell membranes, and the specific ligand engaged—together with local concentrations of sTREM2 that can compete for ligand binding—may shape the downstream signaling output [5, 6]. Similarly, the signaling context matters at the level of the adaptor: signaling through DAP12 recruits Syk, whereas signaling through DAP10 recruits PI3K, and the determinants of coreceptor choice in metabolic tissues remain poorly defined [13, 15]. In HFD‐induced adipose tissue inflammation, TREM2 collaborates with scavenger receptor CD36 to recognize oxidized lipids, promoting macrophage survival via the Syk‐PI3K/Akt pathway, while forming an antagonistic interaction with TLR4 to inhibit NF‐κB‐mediated pro‐inflammatory factor release [9]. In DN under hyperglycemic conditions, TREM2 interacts with the receptor for advanced glycation end products (RAGE): on the one hand, it inhibits macrophage M1 polarization via the DAP12/Syk pathway; on the other hand, it blocks RAGE‐mediated Smad3 phosphorylation to reduce profibrotic factor secretion [10]. In atherosclerotic plaques, TREM2 activation has been reported to upregulate LXRα and enhance the transcription of cholesterol efflux‐related genes (ABCA1 and ABCG1) [37]. The synergistic or antagonistic relationships among these receptors essentially represent the precise programming of TREM2 function by the metabolic microenvironment. By integrating signal inputs related to lipid and glucose metabolism disorders, TREM2 exhibits differentiated functions such as anti‐inflammation, metabolic regulation, or tissue repair in different disease contexts, providing a key molecular perspective for understanding the heterogeneous pathological mechanisms of metabolic diseases.

### 4.3. Regulatory Roles of TREM2 Post‐Translational Modifications (PTMs)

PTMs are critical for regulating TREM2 protein function, stability, and localization. Distinct PTM patterns under different metabolic conditions may represent an important mechanism contributing to TREM2 functional heterogeneity. Protein cleavage is the most well‐characterized modification: persistently elevated pro‐inflammatory factors in NASH activate the metalloproteinase ADAM17, which specifically cleaves the TREM2 ECD to produce sTREM2, leading to reduced membrane‐bound TREM2 and loss of efferocytosis function [9]. In early obesity, however, ADAM17 activity is low, and TREM2 primarily exists in the membrane‐bound form, exerting functions via DAP12 binding [57]. Glycosylation also participates in TREM2 function regulation: studies have confirmed that glycosylation at residue N79 is essential for TREM2 trafficking to the cell membrane, while glycosylation at both N20 and N79 collectively regulates TREM2 intracellular signaling. Additionally, abnormal N‐glycosylation of TREM2 may induce microglial dysfunction, thereby contributing to the pathogenesis of neurodegenerative diseases. It has also been proposed that maintaining TREM2 glycosylation integrity may represent a novel therapeutic direction for such diseases [73]. Collectively, these PTMs further expand the regulatory complexity of TREM2 function regulation and provide potential intervention opportunities for metabolic diseases through the modulation of TREM2 modification processes.

## 5. TREM2‐Targeted Intervention Strategies and Clinical Translation Prospects (Figure 2)

### 5.1. TREM2‐Targeted Intervention Approaches and Research Progress

sTREM2, as an endogenous TREM2‐activating molecule, has demonstrated therapeutic potential in DFU models. In vivo administration of sTREM2 promotes macrophage polarization toward the M2 phenotype and enhances inflammatory resolution, angiogenesis, and collagen deposition at wound sites, thereby accelerating ulcer healing [54]. TREM2 agonistic antibodies (e.g., 4D9) have shown promise in AS models. In advanced atherosclerotic plaques, 4D9 treatment improves plaque stability by enhancing macrophage efferocytosis and reducing necrotic core formation. However, intervention during early‐stage AS may aggravate lesion development by promoting foam cell accumulation, underscoring the critical importance of the disease stage and therapeutic timing [30]. Another TREM2 agonistic antibody, AL002a, has also been reported to reduce necrotic core size and increase fibrous cap thickness and collagen deposition, although these beneficial effects are accompanied by the expansion of overall plaque and macrophage areas [38].

**Figure 2:**
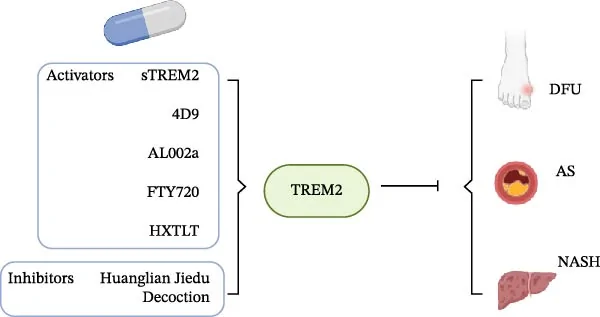
The impact of TREM2 on metabolic diseases. These effects can be mitigated through various interventions, such as sTREM2, 4D9, AL002a, and certain traditional Chinese medicines (TCM).

The S1P–S1PR1 pathway induces TREM2 transcriptional upregulation in hepatic macrophages. In NASH, TREM2 protein is depleted via ADAM17‐mediated ectodomain shedding, resulting in impaired efferocytosis and exacerbated inflammation and fibrosis [9]. Studies have shown that the S1PR1 agonist FTY720 and its derivatives ameliorate NASH‐associated pathological alterations [74].

Nevertheless, direct in vivo evidence demonstrating that activation of the S1P–S1PR1 pathway can restore TREM2 protein levels and reverse TREM2 depletion in NASH models remains unavailable. In a mouse model of NAFLD‐associated sepsis, TREM2 deficiency accelerates the initial NAFLD progression and subsequent sepsis susceptibility. Conversely, TREM2 overexpression in hepatic macrophages improves hepatic energy metabolism and ameliorates sepsis outcomes [71]. However, specific TREM2‐targeted agonists for therapeutic application in this context remain to be developed.

In recent years, traditional Chinese medicine (TCM)‐based interventions targeting TREM2 have emerged as a promising complement to antibody‐ and small‐molecule‐based approaches. In the context of metabolic disease, Huoxue Tongluo Tablets (HXTLT) promote the differentiation of TREM2^+^ macrophages via the PPARγ signaling pathway, enhancing efferocytosis in atherosclerotic lesions, reducing plaque lipid deposition, and improving plaque stability [75]. Notably, in a HFD‐induced obesity model, Huanglian Jiedu Decoction (HLJDD) was reported to alleviate obesity‐associated neuroinflammation and cognitive impairment by inhibiting the TREM2/DAP12/Syk pathway, with limonin identified as the key active component [76]. This seemingly opposing direction of TREM2 modulation, enhanced TREM2 signaling conferring atheroprotection [75] versus TREM2 signaling inhibition improving obesity‐associated CNS outcomes [76], underscores the context‐dependent nature of TREM2 function and the importance of tailoring TCM‐based strategies to the specific pathological context, as discussed in Section 4. Nevertheless, direct evidence for TCM‐mediated TREM2 modulation in metabolic organs (e.g., liver and adipose tissue) remains limited, and systematic mechanistic and translational studies are warranted.

### 5.2. Clinical Translation of TREM2‐Associated Biomarkers

In 2022, Hendrikx et al. [77] validated through mouse models and clinical cohort analyses that circulating sTREM2 levels are significantly elevated in both NASH model mice and NASH patients. These levels correlate with NASH severity, and sTREM2 exhibits superior performance compared with conventional laboratory parameters in discriminating between different stages of fatty liver disease. Furthermore, spatial transcriptomics confirmed that TREM2‐positive macrophages accumulate in liver regions with injury, inflammation, and fibrosis, further corroborating the association between sTREM2 levels and hepatic inflammation/fibrosis and providing mechanistic support for sTREM2 as a diagnostic biomarker for NASH [77]. In another study enrolling 218 patients with elevated liver stiffness, plasma TREM2 levels were approximately doubled in NASH patients, with high diagnostic accuracy. Plasma TREM2 levels correlated with individual histological features of the NAFLD activity score (NAS): steatosis, lobular inflammation, and ballooning, but showed weak correlation with the fibrosis stage. Dual thresholds for inclusion and exclusion were established: a plasma TREM2 level ≤ 38 ng/mL was identified as the optimal exclusion threshold for NASH, while a level ≥ 65 ng/mL was the optimal inclusion threshold. These findings suggest that plasma TREM2 may serve as a reliable biomarker for excluding or identifying NASH with high accuracy, potentially reducing reliance on invasive liver biopsy and facilitating the identification of patients eligible for NASH clinical trials [59]. In AS, studies have reported that circulating sTREM2 correlates with the progression of carotid artery plaques in patients, and TREM2 dysfunction affects plaque stability [30]. Tissue TREM2 expression also holds clinical reference value: TREM2 expression in adipose tissue is positively correlated with BMI in obese patients, with significantly higher levels in males than in females, making it a stratification biomarker for obesity‐related metabolic disorders [56].

In summary, sTREM2 and tissue TREM2 have demonstrated clear value in the diagnosis, disease assessment, and stratification of metabolic diseases such as NASH, AS, and obesity‐related metabolic disorders. Their associations with disease pathological features and supporting mechanistic evidence lay a solid foundation for the clinical translation of TREM2‐associated biomarkers from basic research, holding promise as novel tools for the precise diagnosis and treatment of metabolic diseases.

### 5.3. Challenges and Risks in the Clinical Translation of TREM2‐Targeted Therapies

When considering TREM2 as a therapeutic target, achieving an appropriate balance between therapeutic potential and potential risks is essential. However, the biological complexity of TREM2 signaling indicates that its clinical translation involves challenges far beyond the simple dichotomy of receptor activation versus inhibition. First, TREM2 exhibits highly context‐dependent functions that vary across disease stages and tissue environments. Taking AS as an example, TREM2 activation may contribute to advanced plaque stabilization, whereas it may aggravate early lesion development by promoting foam cell formation [30, 38]. This suggests that the same therapeutic intervention could produce completely opposite outcomes at different stages of the same disease, while the molecular determinants governing this functional transition remain largely unknown. Second, given the broad expression of TREM2 in tissue‐resident macrophages across multiple organs, systemic modulation of TREM2 is likely to affect diverse macrophage populations beyond the intended target tissues. Current delivery approaches lack sufficient organ‐selective specificity, and such inter‐organ functional conflicts make the overall benefit–risk profile of systemic TREM2 modulation difficult to predict. Local administration or tissue‐specific delivery platforms may provide potential solutions to overcome this limitation; however, these strategies remain in the early stages of development. Third, the long‐term consequences of sustained TREM2 activation or inhibition on systemic immune homeostasis have not been comprehensively evaluated. Since macrophage lipid metabolism, phagocytic capacity, and inflammatory resolution are all regulated by TREM2 signaling, prolonged intervention may alter tissue repair capacity and adaptive responses to emerging metabolic stress. Moreover, whether chronic TREM2 modulation induces compensatory alterations in other immune receptors remains unclear. Finally, although sTREM2‐based biomarker strategies have attracted considerable attention, elevated circulating sTREM2 levels may primarily reflect enhanced receptor shedding rather than increased tissue‐level TREM2 activity. Furthermore, the multiorgan origin of circulating sTREM2 limits its specificity as a disease‐specific biomarker. Consistent with this concern, associations between circulating sTREM2 levels and the severity of metabolic diseases have shown considerable variability across different patient cohorts [59, 78], and their clinical utility for patient stratification requires prospective validation. Collectively, the clinical translation of TREM2‐targeted therapies requires careful consideration of intervention timing, tissue specificity, patient stratification, and long‐term safety profiles rather than unconditional therapeutic optimism. Until these fundamental issues are adequately addressed, systemic TREM2 modulation should be regarded as an investigational strategy with an uncertain benefit–risk balance.

## 6. Research Progress in Emerging Interdisciplinary Fields Related to TREM2

### 6.1. TREM2 and the Gut Microbiota–Metabolism Axis

Notably, in 2024, Luccia et al. [79] provided the first evidence linking TREM2 with gut microbial ecology through mouse model studies. They demonstrated that TREM2 deficiency combined with anti‐PD‐1 therapy induces a pro‐inflammatory intestinal immune state characterized by enhanced activation of intestinal macrophages and substantial expansion of *Ruminococcus gnavus* within the gut microbiota. To validate the role of this bacterium, researchers gavaged wild‐type mice with *Ruminococcus gnavus* and wild‐type mice through oral gavage, which enhanced anti‐PD‐1‐mediated tumor control and recapitulated the therapeutic phenotype observed in TREM2‐deficient mice. Further mechanistic analyses revealed that enrichment of *Ruminococcus gnavus* establishes a pro‐inflammatory intestinal microenvironment that promotes the expansion, systemic circulation, and tumor infiltration of TNF‐producing CD4^+^ T cells. Meanwhile, exogenous supplementation of this bacterium reshapes intratumoral macrophages, significantly enhancing the tumor‐shrinking efficacy of PD‐1 inhibitors. This finding uncovers that TREM2 may remotely influence anti‐PD‐1 immunotherapy outcomes through regulation of the intestinal immune microenvironment and gut microbiota composition, providing mechanistic and target support for optimizing immunotherapy through TREM2‐targeted modulation combined with microbiota‐based interventions [79]. Although this study was conducted in the context of cancer immunotherapy, it illustrates a general principle with direct implications for metabolic diseases: TREM2 expressed by tissue macrophages can shape the gut microbiota and, in turn, systemic metabolic and immune homeostasis. Whether similar TREM2‐microbiota circuits operate in metabolic diseases such as obesity and NASH remains to be explored [71].

### 6.2. TREM2 and Interorgan Crosstalk

A second emerging direction concerns the role of TREM2 in interorgan metabolic communication. A representative example is the liver: Hou et al. [71] demonstrated that TREM2 sustains macrophage–hepatocyte metabolic coordination, maintaining hepatic energy supply and protecting against the transition from NAFLD to sepsis‐related liver failure. Within the liver, TREM2^+^ macrophages support hepatocyte function through metabolic coupling, illustrating TREM2‐dependent intercellular communication at the tissue level. Whether analogous TREM2‐mediated crosstalk operates between anatomically distant organs—for example, between adipose tissue and the liver or between the gut and the cardiovascular system—remains to be established. Future studies combining tissue‐specific TREM2 ablation with metabolomics and single‐cell approaches will be required to map the full scope of TREM2‐dependent interorgan signaling in metabolic diseases.

## 7. Unsolved Scientific Questions and Research Prospects

Despite significant progress in understanding the role of TREM2 in metabolic diseases, several critical scientific questions remain to be addressed. (1) The expression profile, cellular localization, and specific regulatory mechanisms of TREM2 in DCM remain incompletely characterized. Further validation using DCM animal models and clinical samples is urgently required to clarify its protective effects and downstream signaling mechanisms. (2) Disease‐stage dependency, activation threshold, and target cell specificity underlying the bidirectional functions of TREM2 in DR remain unclear. Stratified investigations are needed to define stage‐specific therapeutic strategies for different phases of DR progression. (3) The differential regulatory networks governing the synergistic or antagonistic interactions between TREM2 and other immune receptors under distinct metabolic stress conditions, as well as their potential as combined therapeutic targets, warrant further investigation. (4) Major challenges associated with the clinical translation of TREM2‐targeted therapies, including tissue‐specific delivery, optimal therapeutic windows, and long‐term safety profiles, require systematic evaluation through preclinical large‐animal experiments and early‐phase clinical trials.

Future research should focus on the following directions. First, elucidating the context‐dependent and heterogeneous functions of TREM2 across different diseases and immune cell subsets using advanced technologies, including single‐cell sequencing and spatial transcriptomics. Second, developing highly specific selective TREM2‐targeting modulators and precision delivery strategies to improve therapeutic specificity and efficacy. Third, performing multicenter, large‐scale clinical studies to validate the diagnostic and prognostic potential of TREM2‐related biomarkers and facilitate their clinical implementation. Finally, further exploration of the interactions among TREM2, gut microbiota, and epigenetic regulation may provide novel strategies for the comprehensive management of metabolic diseases. In summary, as a central regulatory molecule in the immunometabolic network, TREM2 represents a promising therapeutic and diagnostic target. A deeper understanding of their molecular mechanisms and translational potential in metabolic diseases may open new avenues for precision medicine and the targeted management of chronic metabolic disorders.

## 8. Conclusion

The global prevalence of metabolic diseases and their complex pathological mechanisms represent a major challenge in public health. Dysregulation of the immunometabolic network, a hallmark feature underlying disease progression, provides a critical entry point for understanding disease mechanisms and developing targeted therapies. As a key regulator of immunometabolism, TREM2 exhibits substantial disease‐ and context‐dependent functional complexity in metabolic diseases, and its dysfunction serves as a shared pathogenic mechanism contributing to the progression of multiple metabolic diseases. A comprehensive elucidation of TREM2 regulatory networks may facilitate the discovery of novel biomarkers for precision diagnosis and risk stratification of metabolic diseases, while also providing new opportunities for the development of targeted therapeutic approaches.

## Author Contributions

Conceptualization, data collection, analysis, and writing were performed by Yaling Li and Sheng Quan Liu. Validation, investigation, and supervision were performed by Chun Chu and Jun Yang.

## Funding

This study was supported by the Natural Science Foundation of Hunan Province (Grant 2022JJ40399).

## Disclosure

All authors contributed to the review, commented on previous versions of the manuscript, and read and approved the final manuscript.

## Ethics Statement

This article is based on previously conducted studies and does not contain any studies with human, participants, or animals performed by any of the authors.

## Conflicts of Interest

The authors declare no conflicts of interest.

## Data Availability

Data sharing is not applicable to this article, as no datasets were generated or analyzed during the current study.
